# Molecular Networking-Guided Isolation of a Phenolic Constituent from *Prunus mume* Seed and Its Antioxidant and Anti-Inflammatory Activities

**DOI:** 10.3390/foods12061146

**Published:** 2023-03-08

**Authors:** Chang-Kwon Kim, Jayeon Yu, Mina Lee

**Affiliations:** College of Pharmacy and Research Institute of Life and Pharmaceutical Sciences, Sunchon National University, 255 Jungangno, Suncheon 57922, Jeonnam, Republic of Korea

**Keywords:** *Prunus mume*, molecular networking, ferulic acid, anti-inflammation, antioxidant

## Abstract

*Prunus mume* (Maesil) is used in health foods and alternative medicine in Korea. In the present study, the anti-inflammatory and antioxidant effects of phenolics from *P. mume* seed extracts were examined. First, the biological activities of various *P. mume* extracts were evaluated, and the profiles of their chemical compounds were investigated by Global Natural Products Social (GNPS)-molecular networking. Among these extracts, fermented Maesil seed extract (FMSE) showed potent anti-inflammatory and antioxidant activity, and demonstrated the presence of phenolic clusters in GNPS-based studies. Thus, the chemical constituents of this extract were further investigated. Subsequently, the chemical composition of the active CH_2_Cl_2_ fraction of FMSE was explored using an advanced GNPS analysis tool, MolNetEnhancer. In addition, the molecular structure of compound **1** from the CH_2_Cl_2_ fraction was similarly predicted with Network Annotation Propagation (NAP). Finally, the anti-inflammatory and antioxidant effects of compound **1** were confirmed by lipopolysaccharide (LPS)-induced nitric oxide production and DPPH assay. Western blot analysis revealed that compound **1** downregulated the expression of inducible nitric oxide synthase (iNOS) and cyclooxygenase-2 (COX-2) proteins. The molecular docking simulation additionally confirmed significant interactions of **1** with iNOS and COX-2 proteins. Our findings suggested that an integrated GNPS-based approach could prioritize samples in the early fractionation process and improve the accuracy of target compound prediction.

## 1. Introduction

*Prunus mume* Sieb. et Zucc. (Rosaceae), commonly known as Chinese Plum, Japanese Apricot, and Korean Maesil, is widely cultivated in East Asian countries. It is used as a functional health food and traditional medicine in Korea [1]. Previous studies have reported that *P. mume* contains organic acids, phenolics, terpenes, benzyl glycosides, furfurals, alkaloids, and cyanogenic glycosides [2]. Numerous studies have revealed that *P. mume* has anti-diabetic, anti-tumoral, liver-protective, antimicrobial, antipyretic treatment, hepatoprotective, anti-inflammatory, and antioxidant activities [3,4,5,6,7,8]. The phenolic content of *P. mume* is relatively high, showing potential anti-inflammatory and antioxidant activities [9]. Thus, phenolic compounds from *P. mume* extracts were investigated, based on biological effects and GNPS-based molecular networking, in this study.

Global Natural Product Social (GNPS)-based molecular networking (MN) has emerged as an efficient tool for rapid metabolite identification via untargeted mass spectrometry (MS), or MS-based, data in early research of natural products [10]. This web platform allows the organization and visualization of compounds from natural extracts by comparing annotations obtained from an MS spectral library [11]. Network Annotation Propagation (NAP) and the Mass2Motifs LDA parameter (MS2LDA) can be used to improve the accuracy of candidate prediction and discover unsupervised substructure pattern in MS data [12,13,14]. Outputs from MN, NAP, and MS2LDA can be integrated using MolNetEnhancer workflow, which provides a more extensive chemical overview of metabolomics data through the Classyfire automatic classification module [15,16,17,18].

GNPS-based metabolomic approaches have recently been widely used in food-related research [19,20]. Phytochemicals contained in food material exhibit different kinds of compound classes and contents, influenced by various geographical and environmental factors. With MS/MS-based GNPS approaches, a qualitative or quantitative analysis of these phytochemicals can be performed quickly [21].

In the present study, the profiles of chemical compounds in various seed extracts of *P. mume* and FMSE fractions were investigated by Feature-Based Molecular Networking (FBMN), NAP, MS2LDA, and MolNetEnhancer. The proposed structure of compound **1** in active fraction was predicted by NAP using MetFrag score [22]. Finally, anti-inflammatory and antioxidant activities of compound **1** were evaluated by LPS-induced NO production in macrophages and a DPPH radical scavenging assay. In addition, the possible mechanism of NO inhibition was further supported by western blot analysis and a molecular docking study. The aim of this study was to utilize a GNPS-based approach to prioritize isolation efforts in biologically active compound from *Prunus mume*. The detailed list of abbreviations and acronyms used in the paper are shown in Table 1.

## 2. Materials and Methods

### 2.1. Plant Materials

*Prunus mume* seeds were supplied by korer S-sizipganennarl Cooperatives, Gwangyang-si, Jeollanam-do, Korea, in 2022, and were identified and authenticated by Prof. Mina Lee (College of Pharmacy, Sunchon National University). Their voucher specimens (SCNUP-35) were deposited at the laboratory of Pharmacognosy, College of Pharmacy, Sunchon National University, Suncheon-si, Jeollanam-do, Korea. Sample names were abbreviated as Maesil seed extract (MSE), fermented Maesil seed extract (FMSE), Maesil seed shell extract without oil (MSS), and Fermented Maesil seed shell extract without oil (FMSS), respectively.

### 2.2. Extraction and Isolation

To prepare the screening samples, ground *P. mume* seeds (1 g) were mixed with 10 mL of 100% ethanol and extracted using ultrasonication at room temperature (2 h × 2 cycles). Extracts were filtered through polytetrafluoroethylene (PTFE) membrane filters (Sigma-Aldrich, Saint Louis, MO, USA) and concentrated in vacuum at 39 °C using a rotary evaporator (Eyela, Tokyo, Japan). Finally, concentrated extracts were kept in the shade at 4 °C. For large scale extraction, FMSE (4.0 kg) was extracted with 100% ethanol (6 L) using ultrasonication at rt (2 h × 5 cycles). Extracts were filtered through No.2 Whatman filter paper (Whatman, Pleasanton, CA, USA) and dried under vacuum to obtain 216.9 g of total extract. Subsequently, the extract was then suspended in water and partitioned in a regular sequence with *n*-hexane, CH_2_Cl_2_, EtOAc, and *n*-butanol to obtain 5.9 g, 5.8 g, 7.0 g, and 31.8 g residues, respectively. Among them, the CH_2_Cl_2_ layer showed NO production and DPPH radical scavenging activity. Thus, this layer was used for further isolation. The CH_2_Cl_2_ fraction was separated by preparative reversed-phase MPLC using a Biotage^®^ Sfär C_18_ D column (40 mm × 160 mm; 18.0 mL/min, CH_3_CN-H_2_O gradient (18:82–50:50) containing 0.1% formic acid, detection at 254 nm), yielding seven sub-fractions (MC0-MC6). The retention time and sample amount of the seven sub-fractions were as follows: MC0 (*t*_R_ 0–25 min, 494.1 mg), MC1 (*t*_R_ 25–32 min, 156.7 mg), MC2 (*t*_R_ 32–39 min, 106.1 mg), MC3 (*t*_R_ 39–46 min, 152.3 mg), MC4 (*t*_R_ 47–54 min, 135.3 mg), and MC5 (*t*_R_ 54–61 min, 141.6 mg). Purification of active sub-fraction MC5 by semi-preparative HPLC on a YMC-Triart C_18_ column (10 mm × 250 mm; 3.0 mL/min, CH_3_CN-H_2_O gradient (5:95–30:70) containing 0.1% formic acid, a sample concentration of 5 mg/mL, injection volume of 200 μL, detection at 254 nm) afforded compound **1** (*t*_R_ 42.0 min, 1.5 mg).

### 2.3. DPPH Radical Scavenging Assay

The 2,2-diphenyl-1-picrylhydrazyl (DPPH; Thermo Fisher Scientific, Ward Hill, MA, USA) free radical scavenging activity was measured using a previously reported method [23]. Briefly, a DPPH solution (0.2 mM, 100 μL) was mixed with a sample (100 μL) on a 96-well plate and reacted for 30 min in the shade. The absorbance was measured at 517 nm using a micro reader (Epoch, Biotek Instruments, Inc., Winooski, VT, USA). Ascorbic acid (100 μg/mL) (Sigma-Aldrich, Co., St. Louis, MO, USA) was used as the positive control group. The difference in DPPH radical scavenging activity between the negative control and treated sample was calculated with the following formula: %EC = (A control − A sample) * 100/(A control); A sample, absorbance of the sample; A control, absorbance of untreated sample.

### 2.4. ABTS Radical Scavenging Assay

The 2,2′-azino-bis (3-ethylbenzothiazoline-6-sulfonic acid diammonium salt) (ABTS; Sigma-Aldrich, Co., St. Louis, MO, USA) free radical scavenging activity was evaluated using a previously reported method [24]. Potassium persulfate (2.45 mM) was added to an ABTS (7 mM) solution and reacted at 4 °C for 16 h in the shade. Then the sample (100 µL) and ABTS solution (100 µL) were reacted at room temperature for 6 min in the dark. The absorbance was measured at 734 nm using a micro reader (Epoch, Biotek Instruments, Inc., Winooski, VT, USA). Ascorbic acid (100 µg/mL) was used as the positive control group. The ABTS radical scavenging activity was calculated with the following formula: %EC = (A control − A sample) * 100/(A control); A sample, absorbance of the sample; A control, absorbance of untreated sample.

### 2.5. Cell Culture

RAW 264.7 cells, from a mouse macrophage cell line, were obtained from Korean Cell Line Bank (Seoul, Republic of Korea). These macrophages were grown at 37 °C in a humidified atmosphere with 5% CO_2_. They were maintained in culture medium (Dulbecco’s modified Eagle’s medium (DMEM)) with 10% heat-inactivated fetal bovine serum (FBS), 100 IU/mL Penicillin Solution (HyClone, Logan, UT, USA), and 100 µg/mL Streptomycin.

### 2.6. Cell Viability Assay

Mouse macrophages were plated into 96-well plates at a density of 10^5^ cells/well in DMEM culture medium for 24 h. Cells were mixed with various concentrations of samples for 1 h and then stimulated with LPS (1 µg/mL) for 20 h. Cell viability was evaluated by MTT (3-[4,5-dimethyl-2-thiazolyl]-2,5-diphenyl tetrazolium bromide) (Sigma-Aldrich, Saint Louis, MO, USA) assay. The cultured cells were incubated with MTT (5 mg/mL) at 37 °C for 4 h. The supernatant was then aspirated and 100 µL DMSO was added to each well. After 5 min, the absorbance of the formazan crystals was checked at 570 nm with a micro reader (Bio Tek Instruments, Winooski, VT, USA).

### 2.7. Measurement of NO Production

The cultured mouse macrophages (1 × 10^5^ cells/well) were treated with samples in serum-free culture medium for 2 h and then induced with LPS (1 µg/mL). After 20 h, the supernatant (100 µL) was harvested and mixed with an equal volume of Griess reagent (1% (*w/v*) sulfanilamide in 5% (*v/v*) phosphoric acid and 0.1% (*w/v*) naphtylethylene). After incubation at room temperature for 10 min, the absorbance was measured at 550 nm using a microreader (BioTek Instruments, Inc., Winooski, VE, USA).

### 2.8. Western Blot Analysis

Mouse macrophages were seeded at a density of 1 × 10^6^ cells/well in 6-well plates in DMEM (2 mL) for 24 h. Seeded cells were pretreated with samples for 2 h. Then stimulated with LPS (1 µg/mL) for twenty hours. The cells were washed two times with cold PBS and whole cell lysates were extracted with protein extraction solution (proprep, iNtRON, Biotechnology, Daejeon, Korea) for 30 min. The protein concentration was determined by the Bradford method. A total of 20 μg of mixed protein, water, and 5 × sample buffer (250 mM Tris-HCl (pH 6.8), 10% SDS, 5% 2-mercaptoethanol, 0.2% bromophenol blue, and 50% Glycerol) were heated at 95 °C for 10 min and were separated with 10% SDS-polyacrylamide gels electrophoresis (SDS-PAGE). The separated proteins were transferred to polyvinylidene difluoride (PVDF) membranes. The binding of primary antibodies (iNOS and COX-2, 1: 1000 dilution) and secondary antibodies (mouse IgG for iNOS, COX-2, and β-actin, 1: 1000 dilution) to membranes was evaluated with an iBind western device for three hours. The membranes were washed with buffer solution (4 M NaCl, Tween-20, 1 M Tris-HCl (PH 7.5)) in DW for 10 min and protein signals were obtained using chemiluminescence detection reagents (Thermo Fisher Scientific, Waltham, MA, USA). Bio images were measured with a bio image system (Microchemi 4.2 Chemilumineszenz System, Neve Yamin, Israel)

### 2.9. Statistical Analysis

All data are presented as means ± standard deviations (S.D.) of at least three independent experiments. Nonparametric one-way ANOVA and Dunnett’s multiple comparison test were conducted with Graphprism version 8.0.1 software (GraphPad Software, La Jolla, CA, USA). * *p* < 0.05, ** *p* < 0.01, and *** *p* < 0.001 compared to controls were accepted as statistically significant.

### 2.10. Molecular Networking Analysis

#### 2.10.1. Sample Preparation

*Prunus mume* extracts and CH_2_Cl_2_ fractions of FMSE were diluted with 100% MeOH to a concentration of 30 mg/mL. Sample solutions were filtered with PTFE (polytetrafluoroethylene) membrane filters (Sigma-Aldrich, Saint Louis, MO, USA) for LC-MS/MS analysis.

#### 2.10.2. LC-MS/MS Analysis Conditions

LC-MS/MS analyses were performed on an Orbitrap Exploris 120 MS spectrometer coupled with a Vanquish UHPLC system (Thermo Fisher Scientific, Sunnyvale, CA, USA). Chromatographic separations were conducted with a Waters Acquity UPLC HSS T3 column (4.6 × 100 mm, 1.8 μm, Waters, Milford, MA, USA) at 40 °C with a flow rate of 0.4 mL/min and an injection volume of 4 μL. The mobile phase consisted of a solvent system of phase A (water containing 0.1% formic acid) and phase B (acetonitrile containing 0.1% formic acid), with gradient elution as follows: 5–5% (B) from 0 to 1 min, 5–15% (B) from 1 to 4 min, 15–35% (B) from 4 to 12 min, 35–45% (B) from 12 to 17 min, 45–100% (B) from 17 to 23 min, 100–100% (B) from 23 to 26 min, 100–5% (B) from 26 to 27 min, and 5–5% (B) from 27 to 30 min. The full MS survey scan had *m/z* values between 100 and 1000 Da. The resolution of the Orbitrap mass analyzer was fixed at 60,000 for a full MS scan and 15,000 for a data-dependent mass scan. Heated-Electrospray Ionization (HESI) conditions were set as follows: collision energy (30 V), capillary voltage (2.5 kV) for negative mode, HESI probe vaporizer temperature (275 °C), ion transfer tube temperature (325 °C), and RF lens 70 (%). Ultrapure nitrogen (>99.999%) was used as both the auxiliary and sheath gas of the HESI probe and set to 50 and 15 arb, respectively.

#### 2.10.3. LC-MS/MS Data Analysis

##### MZmine 2.53 Data-Preprocessing Parameters

Raw LC-Orbitrap-MS/MS data files were processed with MZmine 2.53 [25]. Mass detection was performed by keeping noise level at 1.0E3 for MS1 and 1.0E1 for MS2. The chromatogram was built with a minimum time span (0.01 min), a minimum height (3000), and an *m/z* tolerance of 0.0 (or 20.0 ppm). Chromatographic deconvolution was performed used a baseline cutoff algorithm with the following settings: minimum peak height (1500), peak duration range (0.01–3.00 min), baseline level (1000), *m/z* range for MS2 scan pairing of 0.02 Da, and RT range for MS2 scan pairing of 0.1 min. Chromatograms were deisotoped by an isotope peak grouper algorithm at an *m/z* tolerance of 0.0 (or 20.0 ppm) and a *t*_R_ tolerance of 0.1 min. Alignment was conducted with a join aligner module (*m/z* tolerance at 0.0 (or 20.0 ppm), absolute *t*_R_ tolerance at 0.1 min, weight for *m/z* 70, and weight for *t*_R_ 25). The peak list was gap-filled with a peak finder module (intensity tolerance (10.0%), *m/z* tolerance of 0.0 (or 20.0 ppm), and absolute *t*_R_ tolerance at 0.2 min.

##### Feature-Based Molecular Networking

Molecular networking was conducted with the GNPS web platform (https://gnps.ucsd.edu (accessed on 10 January 2023) [26]. Precursor ion and fragment ion tolerance were set to 0.02 Da. The MN was then created using a minimum cosine score above 0.7 and more than six minimum-matched fragment ions and peaks. MS/MS spectra were filtered by choosing only the top six peaks in the ±50 Da window across each spectrum. MS spectral data in the network were then searched for in the spectral library of GNPS. The MN was visualized using Cytoscape 3.9.1 (https://www.cytoscape.org/ (accessed on 1 February 2023)) [27]. The workflow and results of FBMN networking can be found in the following GNPS repository (Task ID = 9748ac424426497392b5dea80fe59d54 and 7e5bb9a58ea34ddea8f53963f11267a9).

##### NAP

Molecular networking was used to confirm the propagation of in silico annotation with NAP [12] using the following parameters: 10 first candidates, exact mass searches within 10 ppm, cosine value to sub-select inside a 0.5 cluster, and GNPS and HMDB structural databases. The workflow and results of NAP can be found in the following GNPS repository (Task ID = 69d5bc6f3d7144319dc2682aff40cdf0 and 200866c8be01478ab2b56abe743436b4).

##### MS2LDA Substructure Discovery

Mass2Motifs were extracted from a pre-processed mgf spectra file [28]. Parameters for the MS2LDA experiment were set as follows: bin width 0.005 Da, number of LDA iterations 1000, minimum MS2 intensity 100, and LDA free motifs 300. The workflow and results of MS2LDA can be found in the following GNPS repository (Task ID = ad2a0a3ca3c04c50b45f74895d606525 and 8879fdbb97194f6582c14d49ab4a91f0).

##### MolNetEnhancer

MolNetEnhancer is a workflow that enables the combination of outputs from FBMN, NAP, and MS2LDA [15]. The workflow and results of MolNetEnhancer can be found in the following GNPS repository (Task ID = 671a7299359847938a9b7633da719a0f and 75e279bd9c5f4b73af25617ffe8124ba).

### 2.11. Molecular Docking

The binding mechanism of compound **1** with iNOS (PDB ID: 1M9T) and COX-2 (PDB ID: 6BL3) proteins was evaluated with a molecular docking simulation. The energy minimization was conducted by Avogadro package via force field method-MMFF94. The protein structure was prepared by the H_2_O molecules and polar hydrogen atoms were added. Kollman charges were assigned to the protein. A grid box with dimensions of 40 × 40 × 40 in 0.375 Å spacing along the x, y, and z axes, respectively, was created. Grid coordinates of center_x = 125.829, center_y = 110.321, and center_z = 32.479 for the iNOS protein (1M9T), and center_x = −41.474, center_y = −28.741, and center_z = 24.872 for the COX-2 protein (6BL3), were used. The docking calculation was conducted with AutoDock Vina software using the MGL tools 1.5.7 program. Each docking experiment was performed for 100 runs. Three-dimensional modeling was performed with PyMOL software (Schrödinger, Inc., New York, NY, USA). A two-dimensional diagram was prepared by Discovery Studio 2021 (BIOVIA, San Diego, CA, USA).

## 3. Results

### 3.1. Antioxidant and Anti-Inflammatory Effects of Various P. mume Extracts

**Figure 1:**
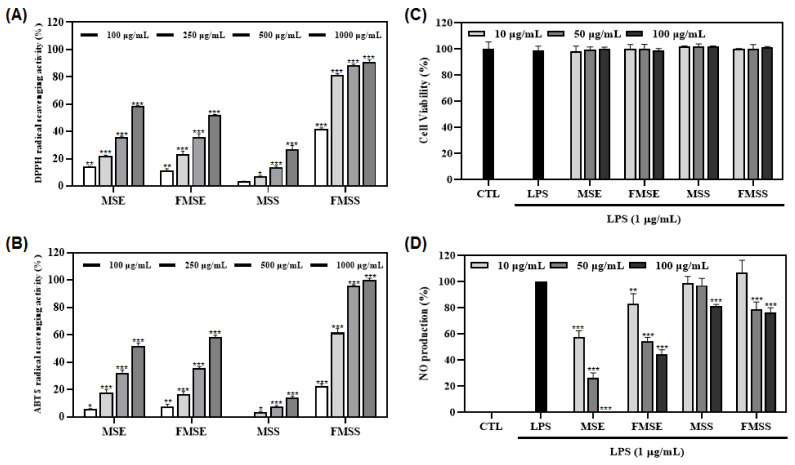
Effects of the various *P. mume* extracts on radical scavenging activities, NO production and cell viability. (**A**,**B**) The antioxidant effects of *P. mume* extracts were evaluated by DPPH (**A**) and ABTS (**B**) radical scavenging assays. The data are expressed as the mean ± SD of three individual experiments. * *p* < 0.05, ** *p* < 0.01, and *** *p* < 0.001 vs. control (blank sample). Ascorbic acid was used as the positive control with an IC_50_ value of (**A**) 13.16 and (**B**) 8.13 µg/mL, respectively. (**C**,**D**) The anti-inflammatory effects of *P. mume* extracts were evaluated by cell viability (**C**) and NO production (**D**) assays. Macrophages were pretreated with *P. mume* extracts at different concentrations (10, 50, and 100 µg/mL) for 1 h and induced with LPS (1 µg/mL) for 20 h. Nitrite concentrations of non-treated and LPS-treated controls were 0.9 ± 0.1 μM and 15.1 ± 0.6 μM, respectively. Cell viability (**C**) and NO production (**D**) were measured using MTT assay and Griess reagent, respectively. Each experiment was conducted in triplicates. The data are represented as mean ± SD. ** *p* < 0.01 and *** *p* < 0.001 vs. LPS-treated group.

Firstly, DPPH and ABTS assays were conducted to investigate the antioxidant capacities of various *P. mume* extracts at 100, 250, 500, and 1000 µg/mL. All *P. mume* extracts exhibited concentration-dependent radical scavenging activities. Among them, the FMSS extract showed the most potent antioxidant activity in both DPPH and ABTS assays (FMSS, 41.8%, MSE, 14.0%, and FMSE, 11.2%, for extracts at 100 µg/mL in the DPPH assay (Figure 1A); FMSS, 22.7%, FMSE, 7.8%, and MSE, 5.6%, for extracts at 100 µg/mL in the ABTS assay (Figure 1B)). Subsequently, the anti-inflammatory effects of various *P. mume* extracts were also evaluated. 

Secondly, all *P. mume* extracts were investigated for their NO-production inhibitory effects in LPS-induced macrophages at different concentrations (10, 50, and 100 µg/mL). Results revealed that the MSE extract showed stronger inhibitory effects (73.7%) on NO production than FMSE (45.3%) and FMSS (20.9%) extracts at 50 µg/mL (Figure 1C). All *P. mume* extracts showed non-cytotoxic effects on cell viability at the same concentrations using MTT assay (Figure 1D). Since different extracts showed good activities in each experiment, molecular networking analysis was further used to perform chemical classification of *P. mume* extracts.

### 3.2. Molecular Networking Analysis of Various P. mume Extracts

**Figure 2:**
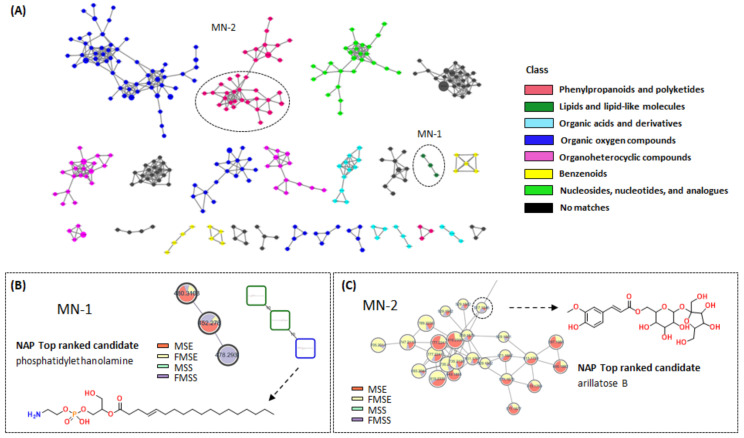
Molecular networking analysis of the various *P. mume* extracts. (**A**) Automatic classification and visualization of each cluster by MolNetEnhancer. (**B**) FBMN and NAP results of the MN-1 cluster. Numbers indicate the precursor ion *m/z*, spectral size indicates the total sum of precursor ion intensity in MS1 scans, and each node is colored according to the different extracts of *P. mume*. The node of *m/z* 478.2938 was predicted by NAP. Node borders in NAP are either green (spectral library match) or blue (in silico prediction). (**C**) FBMN result of MN-2 cluster and NAP top ranked candidate for the node of *m/z* 517.1546.

LC-Orbitrap-MS/MS data of MSE, FMSE, MSS, and FMSS extracts of *P. mume* were analyzed by FBMN via the GNPS web platform (https://gnps.ucsd.edu (accessed on 10 January 2023). A list of candidate compounds with MQ score > 0.7 from the FBMN analysis are included in Supplementary Materials (Table S1). The results of FBMN, NAP, and MS2LDA were integrated by MolNetEnhancer workflow on the GNPS web platform, which automatically classified the chemical class of each cluster (Figure 2A). The entire FBMN, NAP, and MolNetEnhancer results are included in Supplementary Materials (Figures S1–S3). All nodes were arranged into multiple classes, containing “Phenylpropanoids and polyketides”, “Organic oxygen compounds”, “Organoheterocyclic compounds”, “Organic acids and derivatives”, “Benzenoids”, and so forth. Among them, the MN-1 cluster (lipids and lipid-like molecules), the precursor ion of *m/z* 478.2938 [M-H]^−^, was identified as phosphatidylethanolamine (PE, 18:1/0:0) by NAP (Figure 2B). The content of this node was further analyzed by FBMN. As a result, it was revealed that the FMSS extract contained highly lipid-like molecules in the MN-1 cluster (Figure 2B) and base peak ion (BPI) MS chromatograms (Figure S4). Thus, the FMSS extract was excluded, to focus on the isolation of phenolic compounds.

Finally, to select material between MSE and FMSE, the obtained yields of FMSE (6.2%) and MSE (5.6%) were initially compared. Additionally, in the molecular networking analysis, the FMSE extract (34.7%) showed higher content than MSE (33.1%) in the MN-2 cluster categorized as phenylpropanoids and polyketides (Figure 2C). Thus, the FMSE extract was finally selected for further study.

### 3.3. Antioxidant Activities of Fractions and Sub-Fractions of FMSE Extract

**Figure 3:**
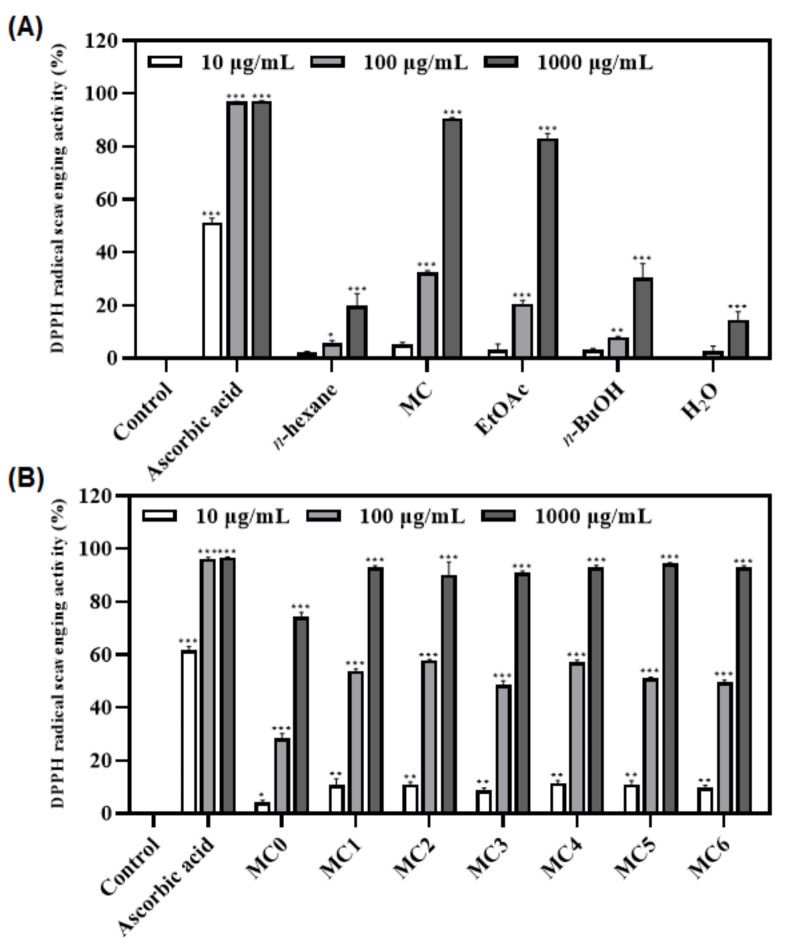
Effects of the fractions (**A**) and sub-fractions (**B**) of FMSE extract on DPPH radical scavenging assays. The data are shown as the mean ± SD (n = 3) of three individual experiments. * *p* < 0.05, ** *p* < 0.01, and *** *p* < 0.001, compared with control (blank sample). Ascorbic acid was used as the positive control.

The 100% EtOH extract of FMSE was partitioned into *n*-hexane, CH_2_Cl_2_, EtOAc, *n*-BuOH, and H_2_O fractions. Antioxidant activities of these fractions were evaluated by DPPH assay. Results indicated that the MC (CH_2_Cl_2_) fraction showed the highest (32.4%) radical scavenging activity at 100 µg/mL in DPPH assay (Figure 3A). Subsequently, sub-fractions (MC0-MC6) obtained from the MC fraction were also evaluated by DPPH assay. Among them, MC2 (57.9%), MC4 (57.1%), and MC5 (51.0%) showed similar potent radical scavenging activities at 100 µg/mL (Figure 3B). Thus, the anti-inflammatory effects of FMSE fractions and sub-fractions were evaluated by further study. 

### 3.4. Anti-Inflammatory Activities of Fractions and Sub-Fractions of FMSE Extract

**Figure 4:**
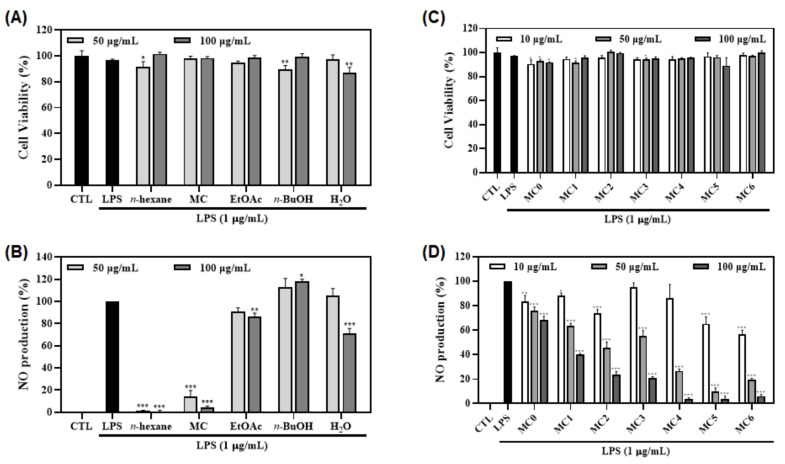
Effects of the fractions and sub-fractions of FMSE on cell viability and NO production. (**A***–***D**) Macrophages were pretreated with the fractions and sub-fractions of FMSE (50 and 100 µg/mL and 10, 50, and 100 µg/mL, respectively) for 1 h and induced with LPS (1 µg/mL) for 20 h. Nitrite concentrations of non-treated and LPS-treated controls were 1.0 ± 0.1 μM and 10.1 ± 0.3 μM (**B**) and 0.9 ± 0.1 μM and 8.8 ± 0.04 μM (**D**), respectively. Cell viability (**A**,C) and NO production (**B**,**D**) were measured with MTT assay and Griess reagent, respectively. Each experiment was performed in triplicates. The data are represented as mean ± SD. * *p* < 0.05, ** *p* < 0.01, and *** *p* < 0.001 vs. LPS-treated group.

The anti-inflammatory activities of these fractions were evaluated with LPS-induced NO production and cytotoxicity. Results indicated that none of them showed a cytotoxic effect (cell viability > 95%) (Figure 4A,C). The MC fraction exhibited a potent inhibitory activity (96% inhibition on NO production as compared to the LPS group at 100 µg/mL) (Figure 4B). Sub-fractions (MC0-MC6) were also examined for their inhibitory effects on NO production in LPS-stimulated macrophages using different concentrations (10, 50, and 100 µg/mL). Among them, at 50 µg/mL, MC5 (89.9%) showed the most potent inhibitory activity, followed by MC6 (80.7%) and MC4 (73.4%) (Figure 4D). Thus, chemical constituents of this fraction were further investigated in combination with molecular networking analysis.

### 3.5. Molecular Networking Analysis of FMSE Extract, MC Fraction, and MC5 Subfraction

#### 3.5.1. Feature-Based Molecular Networking Analysis of FMSE Extract, MC Fraction, and MC5 Subfraction

**Figure 5:**
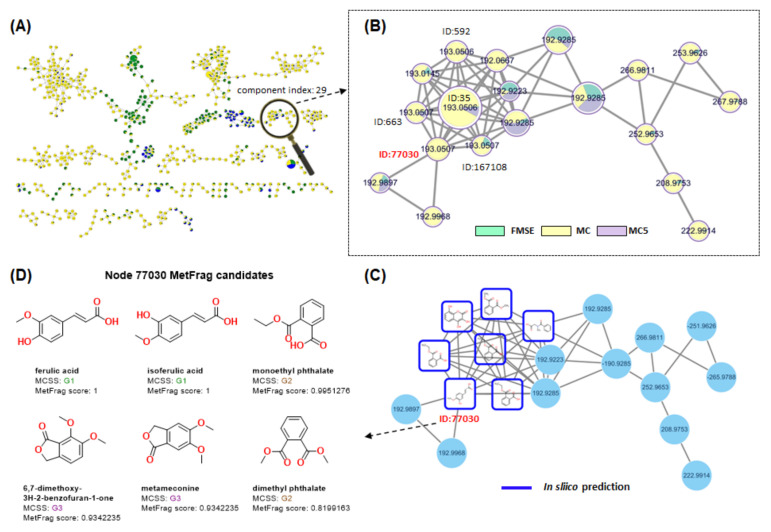
Feature-based molecular networking and NAP analysis of FMSE extract, MC, and MC5 fractions. (**A**) Molecular network of all the detected features (three or more connected nodes of a graph). (**B**) Expanded for component index 29 in the molecular network. Feature IDs were indicated for *m/z* = 193.0506 or 193.0507 [M-H]^−^ of precursor ions. (**C**) NAP result of component index 29, in silico prediction (blue squared). (**D**) Structures of top ranked NAP candidates for node 77030 by MCSS-based algorithm.

The active MC5 fraction was further isolated by semi-preparative HPLC on a YMC-Triart C_18_ column (10 mm × 250 mm; 3.0 mL/min, CH_3_CN-H_2_O gradient (5:95–30:70), detection at 254 nm), yielding compound **1**. Subsequently, to find the molecular cluster of compound **1**, FBMN analysis was conducted using the FMSE extract, MC fraction, and MC5 subfraction. FBMN organized them into a network consisting of 54 molecular families (three or more connected nodes of a graph) (Figure 5A). The entire FBMN result is included in Supplementary Materials (Figure S5). Among them, a molecular cluster (component index: 29) containing *m/z* = 193.0506 or 193.0507 [M-H]^−^ (precursor ion of **1**), which indicated five different nodes (feature ID: 35, 592, 663, 77030, and 167108) (Figure 5B), was discovered. To distinguish the node of compound **1**, their retention times were compared (feature ID: 35, 592, 663, 77030, and 167108, *t*_R_ = 9.5, 8.7, 11.2, 6.9, and 6.2 min, respectively). As a result, it was confirmed that feature ID: 77030 had the same retention time as compound **1**. Subsequently, NAP analysis was conducted to perform in silico chemical structure annotation.

#### 3.5.2. NAP Analysis of FMSE Extract, MC Fraction, and MC5 Subfraction

NAP analysis was performed using the FMSE extract, MC fraction, and MC5 subfraction. The entire NAP result is included in Supplementary Materials (Figure S6). Results of the NAP analysis revealed seven nodes through in silico prediction (blue squared) (Figure 5C). Among them, node 77030 was predicted to have six candidate structures (ferulic acid, isoferulic acid, dimethyl phthalate, 6,7-dimethoxy-3H-2-benzofuran-1-one, metamconine, and dimethyl phthalate) by MetFrag score combined with the maximum common substructure (MCSS)-based algorithm (Figure 5D) [12]. The detailed NAP viewer result for node 77030 is included in Supplementary Materials (Figure S7). Finally, the structure of compound **1** was expected to be ferulic acid or isoferulic acid that possessed the same substructure as MetFrag score 1.

#### 3.5.3. MS2LDA and MolNetEnhancer Analysis of FMSE Extract, MC Fraction, and MC5 Subfraction

**Figure 6:**
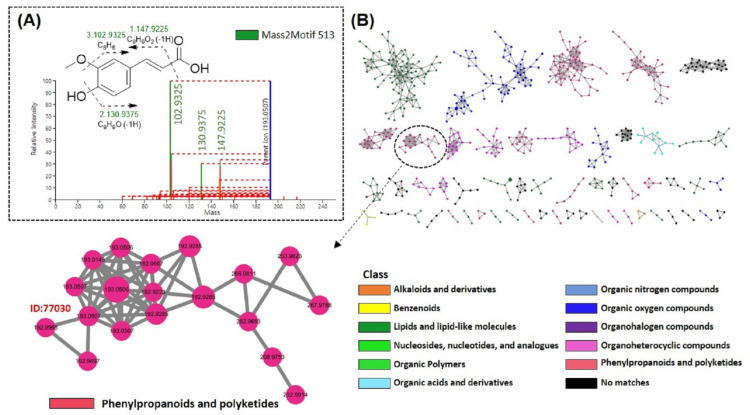
MS2LDA and MolNetEnhancer analysis of FMSE extract, MC, and MC5 fractions. (**A**) MS2LDA-driven fragment patterns of the top ranked NAP candidate. (**B**) Molecular network of all the detected features (three or more connected nodes of a graph), expanded for component index 29 containing feature ID 77030.

Mass2Motif (mass fragments) 513 associated with node 77030 represented the presence of fragment ions *m/z* 102.9325 [C_8_H_6_]^−^, 130.9375 [C_9_H_9_O]^−^, and 147.9225 [C_9_H_9_O_2_]^−^, caused by the loss of methoxyl, hydroxyl, and carboxylic acid moieties, respectively (Figure 6A). Based on these fragment patterns, node 77030 could be annotated as hydroxycinnamic acid-related compounds. Subsequently, outputs from FBMN, NAP, and MS2LDA were integrated by MolNetEnhancer. The entire MolNetEnhancer result is included in Supplementary Materials (Figure S8). Among a total of 54 molecular families (three or more connected nodes of a graph), 13 were “phenylpropanoids and polyketides”, 12 were “lipids and lipid-like molecules”, 6 were “organoheterocyclic compounds”, 4 were “organic oxygen compounds”, 1 was “organic acids and derivatives”, 1 was “alkaloids and derivatives”, 1 was “benzenoids”, and 16 were “no matches”. The molecular family containing node 77030 was annotated as phenylpropanoids and polyketides in the entire network (Figure 6B).

### 3.6. Structure Identification of Ferulic Acid by NMR Analysis

The structure of compound **1** between ferulic acid and isoferulic acid was finally determined by NMR analysis. Its ^1^H NMR spectrum (CD_3_OD) revealed the presence of one typical aromatic ABX spin system with signals at δ_H_ 7.16 (1H, d, *J* = 1.9 Hz, H-2), 7.04 (1H, dd, *J* = 8.4, 1.9 Hz, H-6), and 6.78 (1H, d, *J* = 8.4 Hz, H-5), as well as one *trans* double bond with signals at *δ*_H_ 7.53 (1H, d, *J* = 15.8 Hz, H-7) and 6.32 (1H, d, *J* = 15.9 Hz, H-8). A connection between H-2 and H-7 was confirmed by HMBC correlation from *δ*_H_ 7.53 (H-7) to *δ*_C_ 111.3 (C-2). Additionally, hydoroxyl and methoxyl group positions were confirmed by the following HMBC correlations: H-7/C-2, H-2, and H-6/C-4, and H-5 and H-10/C3, respectively (Supplementary Materials, Figure S9). Thus, the structure of compound **1**, ferulic acid, was identified based on spectroscopic analyses and comparison of spectroscopic data with the reported compound [29]. The ^1^H, ^13^C, and 2D NMR spectra of compound **1** are included in Supplementary Materials (Figures S10–S13). 

#### Spectroscopic Data of Ferulic Acid

Ferulic acid (**1**): white amorphous powder; ESI-HRMS: 193.0508 [M-H]^−^ (C_10_H_9_O_4_); ^1^H NMR (CD_3_OD, 400 MHz): δ 7.53 (1H, d, *J* = 15.8 Hz, H-7), 7.16 (1H, d, *J* = 1.9 Hz, H-2), 7.04 (1H, dd, *J* = 8.4, 1.9 Hz, H-6), 6.78 (1H, d, *J* = 8.4 Hz, H-5), 6.32 (1H, d, *J* = 15.8 Hz, H-8), 3.89 (3H, s, H-10); ^13^C NMR (CD_3_OD, 100 MHz): δ 171.0 (C-9), 150.1 (C-4), 149.3 (C-3), 145.4 (C-7), 128.1 (C-1), 123.7 (C-6), 117.3 (C-8), 116.2 (C-5), 111.3 (C-2), 56.4 (C-10).

### 3.7. Antioxidant and Anti-Inflammatory Effects of Ferulic Acid (***1***)

**Figure 7:**
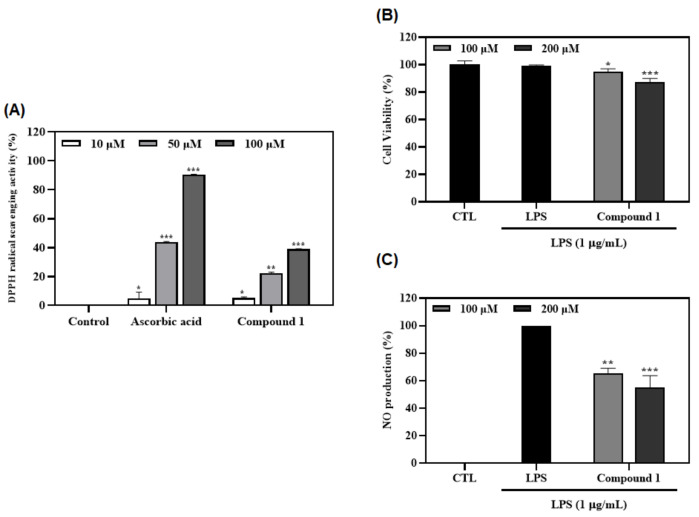
Effects of the compound **1** on radical scavenging activity, cell viability, and NO production. (**A**) The antioxidant effect of compound **1** was evaluated by DPPH radical scavenging assay. The data are expressed as the mean ± SD of three individual experiments. * *p* < 0.05, ** *p* < 0.01, and *** *p* < 0.001 vs. control (blank sample). Ascorbic acid was used as the positive control. (**B**,**C**) The anti-inflammatory effect of compound **1** was evaluated by cell viability (**B**) and NO production (**C**) assays. Macrophages were treated with compound **1** (100 µM and 200 µM) for 1 h and stimulated with LPS (1 µg/mL) for 20 h. Nitrite concentrations of non-treated and LPS-treated controls were 1.2 ± 0.1 μM and 7.5 ± 0.4 μM, respectively. Cell viability (**B**) and NO production (**C**) were conducted using MTT assay and Griess reagent, respectively. The data are expressed as the mean ± SD (n = 3) of three individual experiments. * *p* < 0.05, ** *p* < 0.01, and *** *p* < 0.001, compared with control (blank sample).

Antioxidant activity of compound **1** was evaluated by DPPH assay at concentrations of 10, 50, and 100 µM. Results revealed that compound **1** showed potent DPPH radical scavenging activity (39.1%) at a concentration of 100 µM (Figure 7A). To confirm the anti-inflammatory effect of compound **1** (100 and 200 µM), macrophages were pretreated with compound **1** for 2 h and then stimulated with LPS (1 µg/mL) for 20 h. Control groups were treated with or without LPS in the absence of the sample. A total of 100 µL of the supernatant was harvested and NO production was measured with Griess reagent. Subsequently, cell viability was checked using MTT assay. As a result, compound **1** showed no cytotoxicity. It inhibited NO production in LPS-stimulated macrophages (by 44.8%) at a concentration of 200 µM compared with the control (*p* < 0.001) (Figure 7B,C).

### 3.8. Effects of the Compound ***1*** on iNOS and COX-2 Expression

**Figure 8:**
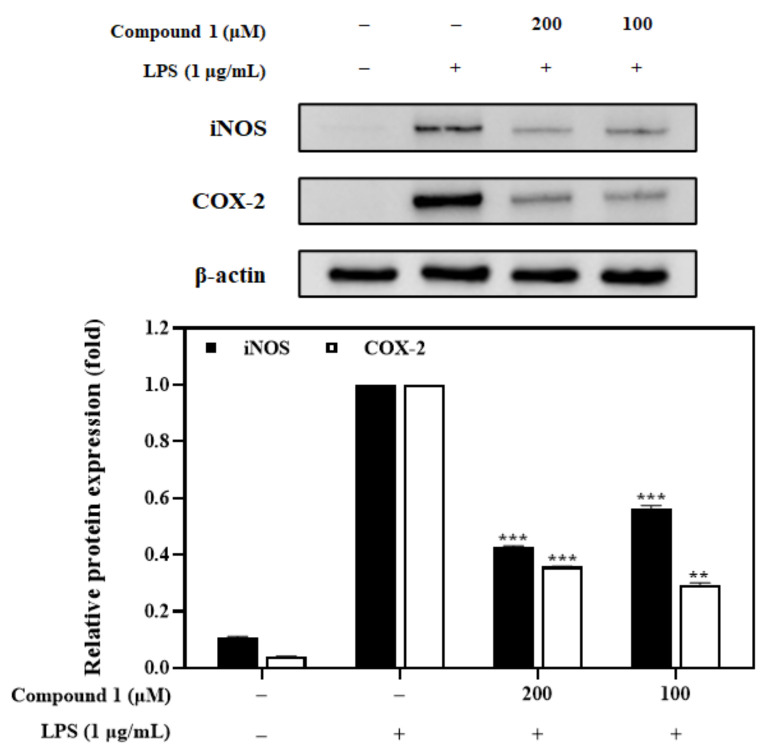
Effects of compound **1** on iNOS and COX-2 expression. Macrophages were incubated in the presence of compound **1** (100 and 200 µM) for 2 h and induced with LPS (1 µg/mL) for 20 h. The levels of iNOS, COX-2, and β-actin in the LPS-induced cells were analyzed by western blot analysis. Relative density was calculated as the ratio of the expression level of each protein with β-actin. Data are represented as mean ± SD. ** *p* < 0.01 and *** *p* < 0.001 vs. LPS-treated group.

Western blot analysis was carried out to investigate the anti-inflammatory effect of compound **1** on the expression of iNOS and COX-2 in RAW 264.7 cells. Results indicated that pretreatment with compound **1** (100 and 200 μM) exhibited a concentration-dependent inhibition of LPS-induced iNOS and COX-2 proteins (Figure 8).

### 3.9. Molecular Docking

**Figure 9:**
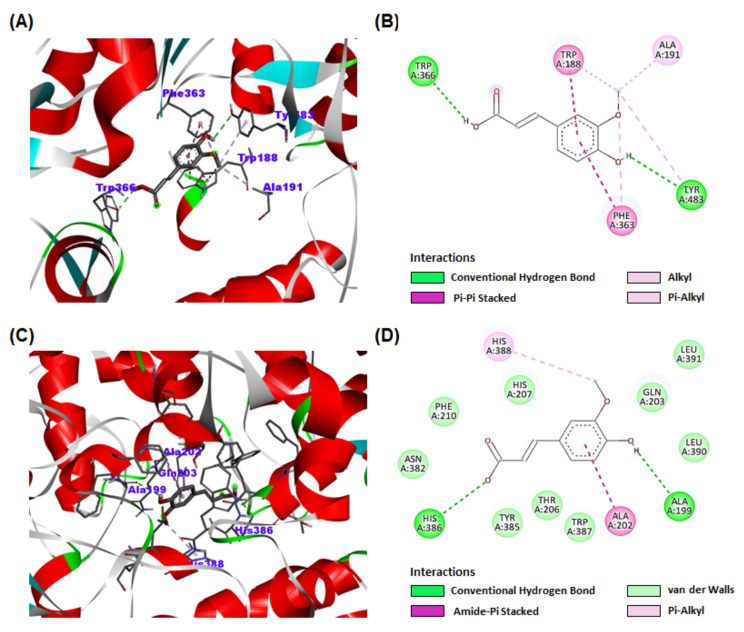
Molecular docking simulations of compound **1** binding to iNOS and COX-2 proteins. (**A**,**C**) Three-dimensional modeling of compound **1** binding within iNOS and COX-2 proteins. (**B**,**D**) Two-dimensional ligand interaction between compound **1** and iNOS and COX-2 proteins.

Molecular docking was further carried out to investigate the possible mechanism of NO inhibition and the interactions of compound **1** with the iNOS (PDB ID: 1M9T) and COX-2 proteins (PDB ID: 6BL3). Results indicated that compound **1** bound in the catalytic pocket of iNOS and COX-2 with an affinity score of −6.8 and −7.2 kcal/mol, respectively (Figure 9A,C). Hydrogen bond interactions between compound **1** and iNOS (TRP366 and TYR483) and COX-2 (HIS386 and ALA199) were proposed as the key contribution to the inhibitory activities (Figure 9B,D).

## 4. Discussion

In the past few years, tandem mass spectrometry (MS)/MS-based molecular networking has proven to be a promising approach to the rapid identification of complex natural product mixtures [30]. GNPS-based molecular networking (MN) allows the organization and visualization of raw tandem MS/MS data sets and automatic searching for specialized metabolite using the database [31]. In addition, advanced data analysis tools, such as FBMN, NAP, MS2LDA, and MolNetEnhancer, enable us to access more exact chemical annotation, classification, and discoveries of substructure diversity [32]. Through these analysis tools, the chemical constituents contained in *P. mume* extracts were rapidly analyzed. 

Before MS/MS analysis, biological activities of various *P. mume* extracts were evaluated. As a result, the FMSS extract showed the most potent antioxidant activity in both DPPH (41.8%) and ABTS (22.7%) experiments (Figure 1A,B), whereas the MSE extract (73.7%) indicated better NO inhibition activity than other extracts (Figure 1D). The antioxidative and anti-inflammatory effects of *P. mume* extract were also found in previous studies [33,34]. Since different extracts showed good activities in each experiment, molecular networking analysis was further conducted to perform chemical classification of *P. mume* extracts. 

Molecular networking provides a convenient way to fully investigate each metabolite by approaching its precursor and parent mass, molecular formula, chromatographic retention times, sum (precursor intensity), and MS/MS spectral properties [35]. In this present study, various *P. mume* extracts were quickly analyzed by FBMN, NAP, MS2LDA, and MolNetEnhancer via the GNPS web platform. As a result, the FMSS extract showed higher content of MN-1 clusters categorized as lipids and lipid-like molecules than other extracts (Figure 2B), whereas the FMSE extract showed higher content (34.7%) of phenylpropanoids and polyketides (MN-2 cluster) than MSE (33.1%) (Figure 2C). To focus on the isolation of phenolic compounds, the FMSE extract was finally selected for further study.

The FMSE extract was then partitioned into *n*-hexane, MC, EtOAc, *n*-BuOH, and H_2_O fractions. In the DPPH assay, the MC fraction showed the most potent antioxidant activity (Figure 3A), and MC2, MC4, and MC5 (51.0–57.1%) indicated similar radical scavenging activities in the sub-fraction tests (Figure 3B). Thus, anti-inflammatory activity was further evaluated. As a results, the MC fraction also showed a potent inhibitory effect (96% inhibition at 100 µg/mL) on NO production (Figure 4B), and MC5 (89.9%) showed the most potent inhibitory activity (Figure 4D) in the sub-fractions test. Thus, MC5 fraction was finally selected. Subsequently, chemical constituents of this fraction were investigated by molecular networking analysis.

Compound **1,** obtained from the MC5 fraction, *m/z* = 193.0506 [M-H]^−^, was discovered in the (Index No: 29) molecular cluster by FBMN analysis (Figure 5A). FBMN enables quantitative analysis, the identification of isomers, and a more exact statistical evaluation of datasets [36]. Through this analysis, five different nodes (feature ID: 35, 592, 663, 77030, and 167108), indicating *m/z* = 193.0506 or 193.0507 [M-H]- (precursor ion), were discovered. Among them, compound **1** was identified as feature ID 77030 by comparison of retention times (Figure 5B).

Subsequently, a NAP experiment was conducted to predict candidate structures for ID 77030 (Figure 5C). NAP analysis was performed with the in silico fragmentation tool MetFrag, which calculates a score that indicates how well the candidate could match a given MS/MS spectrum [37]. Top ranked candidate structures were predicted and the results revealed that ID 7030 could be ferulic acid or isoferulic acid, by MetFrag score 1 (Figure 5D).

The MS2LDA tool enables the discovery of unsupervised groups of neutral losses and mass fragments, called Mass2Motifs, which can show how compounds in the same chemical class are different in their substructures [38]. Through this analysis tool, fragment patterns for candidate structures (feature ID: 77030) were additionally confirmed (Figure 6A). Finally, the structure of ferulic acid (**1**) was further confirmed by NMR analysis (Figure S9).

The antioxidant and anti-inflammatory effects of ferulic acid (**1**) were confirmed by DPPH radical scavenging activity (39.1%) at a concentration of 100 µM (Figure 7A), and NO production activity in macrophages (by 44.8%) at a concentration of 200 µM (Figure 7B,C), respectively. The antioxidant and anti-inflammatory effects of ferulic acid (**1**) can also be found in the following literature [39,40]. To confirm the possible mechanism of NO inhibition, the effects of compound **1** on iNOS and COX-2 expressions were examined. These results demonstrated that compound **1** decreased the expression of iNOS and COX-2 in a concentration-dependent manner (Figure 8). Furthermore, to evaluate binding affinity with iNOS and COX-2 proteins, a molecular docking study was conducted. Results revealed that compound **1** has significant interactions with iNOS and COX-2 proteins, with affinity scores of -6.8 and -7.2 kcal/mol, respectively (Figure 9A,C). To the best of our knowledge, this is the first docking simulation of ferulic acid (**1**) with iNOS (PDB ID: 1M9T) and COX-2 (PDB ID: 6BL3).

Ferulic acid (**1**) is a hydroxycinnamic acid which has polar groups, an aromatic ring, and methoxyl group. These unique structural features enable compound **1** to have both hydrophilic and hydrophobic interactions with the residues at the binding sites of the iNOS and COX-2 proteins. These results strongly suggest the potent anti-inflammatory effect and therapeutic efficacy of compound **1**. Ferulic acid is also considered to be an important antioxidant because its radicals are quickly stabilized by resonance stabilization [41]. It is absorbed into the body relatively faster and stays in the blood longer than other phenolic compounds [42]. Hence, it has been widely used in the food, pharmaceutical, and other industries. Our results further confirmed its antioxidative effects.

Natural products are the richest sources of chemical compounds for improving health. However, chemical profiling of a sample remains challenging, requiring the identification of bioactive compounds from complex extracts [43]. To circumvent this issue, artificial intelligence technology was developed in NPs research. MS/MS-based GNPS molecular networking can produce visual networks consisting of chemically associated compounds and enables the identification of known compounds, structural class, and analogs [44]. This study revealed that a combination of bioactivity evaluation with an MS/MS-based GNPS approach can allow us to prioritize various *Prunus mume* extracts in the early fractionation process and improve the accuracy of target compound prediction.

## 5. Conclusions

In conclusions the antioxidant and anti-inflammatory effects of *P. mume* seed extracts and FMSE fractions were evaluated by performing DPPH and ABTS radical scavenging assays and a NO production assay using LPS-induced macrophages. In addition, to search for phenolic constituents from *P. mume*, MS/MS-based GNPS-molecular networking was applied. As a results, the MC fraction in FMSE showed potent inhibitory activities and high phenolic contents. It subsequently afforded compound **1** via isolation, and was analyzed by advanced GNPS analysis tools such as FBMN, NAP, MS2LDA, and MolNetEnhancer. Finally, the antioxidant and anti-inflammatory effects of ferulic acid (**1**) were confirmed by DPPH radical scavenging assay and NO production assay using LPS-induced macrophages. Furthermore, western blot analysis and molecular docking study on iNOS and COX-2 proteins revealed that compound **1** has strong interactions with these proteins. In summary, combining MS/MS-based GNPS approaches with bioactivity evaluation can help us prioritize samples for further investigation and reinforce the identification of specific chemical classes and compounds. These tools are continuously being developed and will impact the field of natural product research over the next few years.

## Figures and Tables

**Table 1:** List of abbreviations and acronyms used in the paper.

Abbreviation	Definition	Abbreviation	Definition
*P. mume*	*Prunus mume*	FMSE	Fermented Maesil Seed Extract
GNPS	Global Natural Products Social	MSE	Maesil Seed Extract
MN	Molecular Networking	MSS	Maesil Seed Shell extract without oil
MS2LDA	Mass2Motifs LDA parameter	FMSS	Fermented Maesil Seed Shell extract without oil
MolNetEnhancer	Enhanced Molecular Networks	UPLC	Ultra Performance Liquid Chromatography
NAP	Network Annotation Propagation	HMDB	Human Metabolome Database
LPS	Lipopolysaccharide	PDB	Protein Data Bank
DPPH	1,1-diphenyl-β-picrylhydrazine	MCSS	Maximum Common Substructure
ABTS	3-ethylbenzothiazoline-6-sulfonic aciddiammonium salt	COX-2	Cyclooxygenase-2
NO	Nitric Oxide	iNOS	Inducible Nitric Oxide Synthase

## Data Availability

Data is contained within the article or Supplementary Material.

## Supplementary Materials

The following supporting information can be downloaded at https://www.mdpi.com/article/10.3390/foods12061146/s1: Figure S1: The entire feature-based molecular networking analysis of various *P. mume* extracts. Numbers were indicated precursor ion *m*/*z*, spectral size were indicated total sum of precursor ion intensity, each nodes were colored according to the different extracts of *P. mume*; Figure S2: The entire NAP result of various *P. mume* extracts. Node borders in NAP are either green (spectral library match) or blue (in silico prediction); Figure S3: The entire MolNetEnhancer result of various *P. mume* extracts; Figure S4: UPLC-UV-Orbitrap MS chromatograms of various *P. mume* extracts; Figure S5: The entire feature-based molecular networking analysis of FMSE extract, MC fraction, and MC5 subfraction; Figure S6: The entire NAP result of FMSE extract, MC fraction, and MC5 subfraction. Node borders in NAP are either green (spectral library match) or blue (in silico prediction); Figure S7: NAP viewer result for node 77030; Figure S8: The entire MolNetEnhancer result of FMSE extract, MC fraction, and MC5 subfraction; Figure S9: The planar structure and 2D NMR correlations of compound **1**; Figure S10: ^1^H NMR (400 MHz, MeOH-*d*_4_) spectrum of **1**; Figure S11: ^13^C NMR (100 MHz, MeOH-*d*_4_) spectrum of **1**; Figure S12: HSQC NMR (400 MHz, MeOH-*d*_4_) spectrum of **1**; Figure S13: HMBC NMR (400 MHz, MeOH-*d*_4_) spectrum of **1**; Table S1: Compounds list of candidates with MQ score > 0.7 from the FBMN networking analysis at GNPS.
