# Supplementary material for: Molecular Networking-Guided Isolation of a Phenolic Constituent from Prunus mume Seed and Its Antioxidant and Anti-Inflammatory Activities
Source: Foods. 2023 Mar 8;12(6):1146. doi: 10.3390/foods12061146 (PMC10048313; doi:10.3390/foods12061146)
Supplement: Supplementary file 1 [file foods-12-01146-s001.zip › foods-2243295-supplementary.pdf]

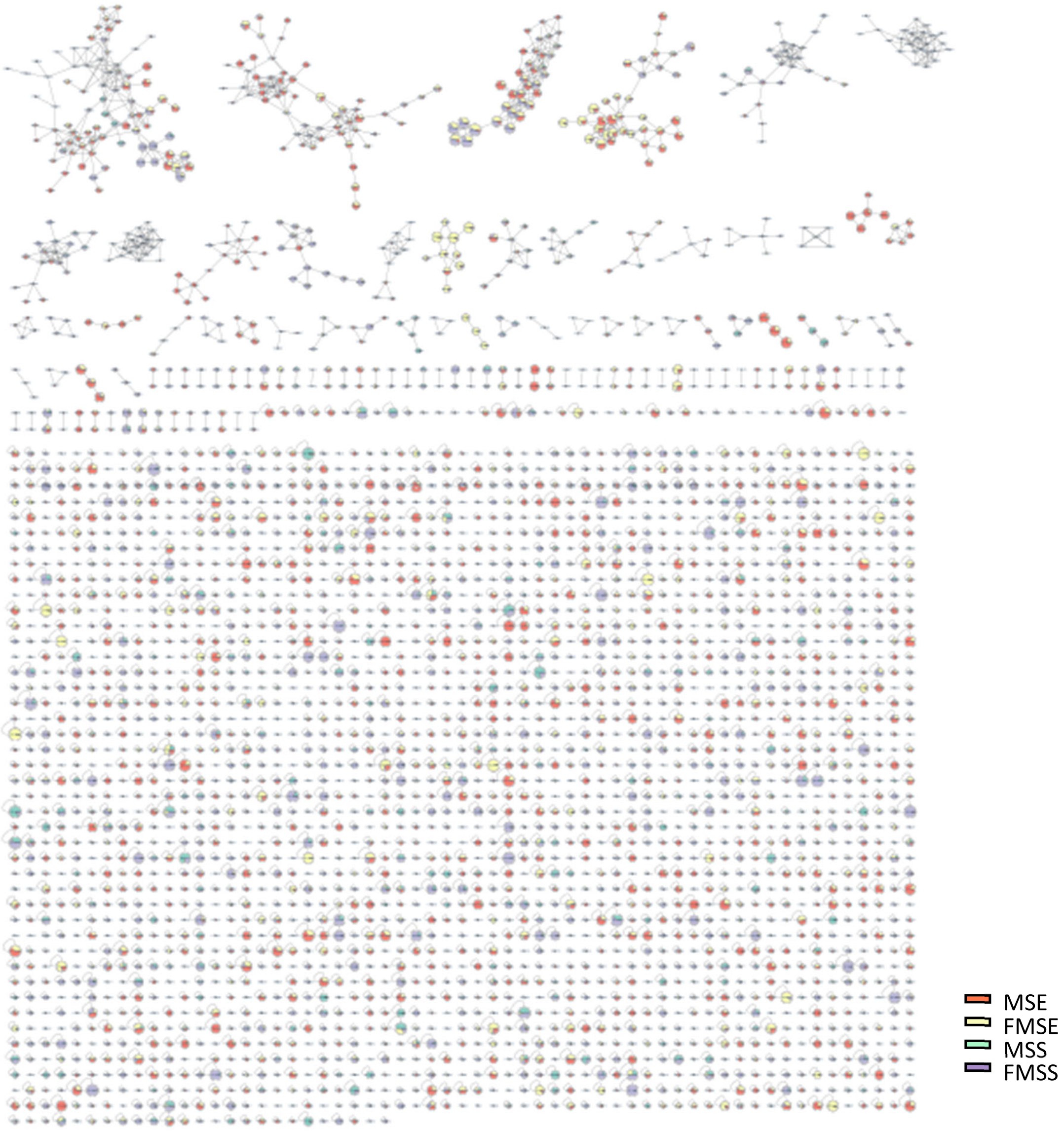

Figure S1. The entire feature-based molecular networking analysis of various *P. mume* extracts. Numbers were indicated precursor ion m/z, spectral size were indicated total sum of precursor ion intensity, each nodes were colored according to the different extracts of *P. mume*.

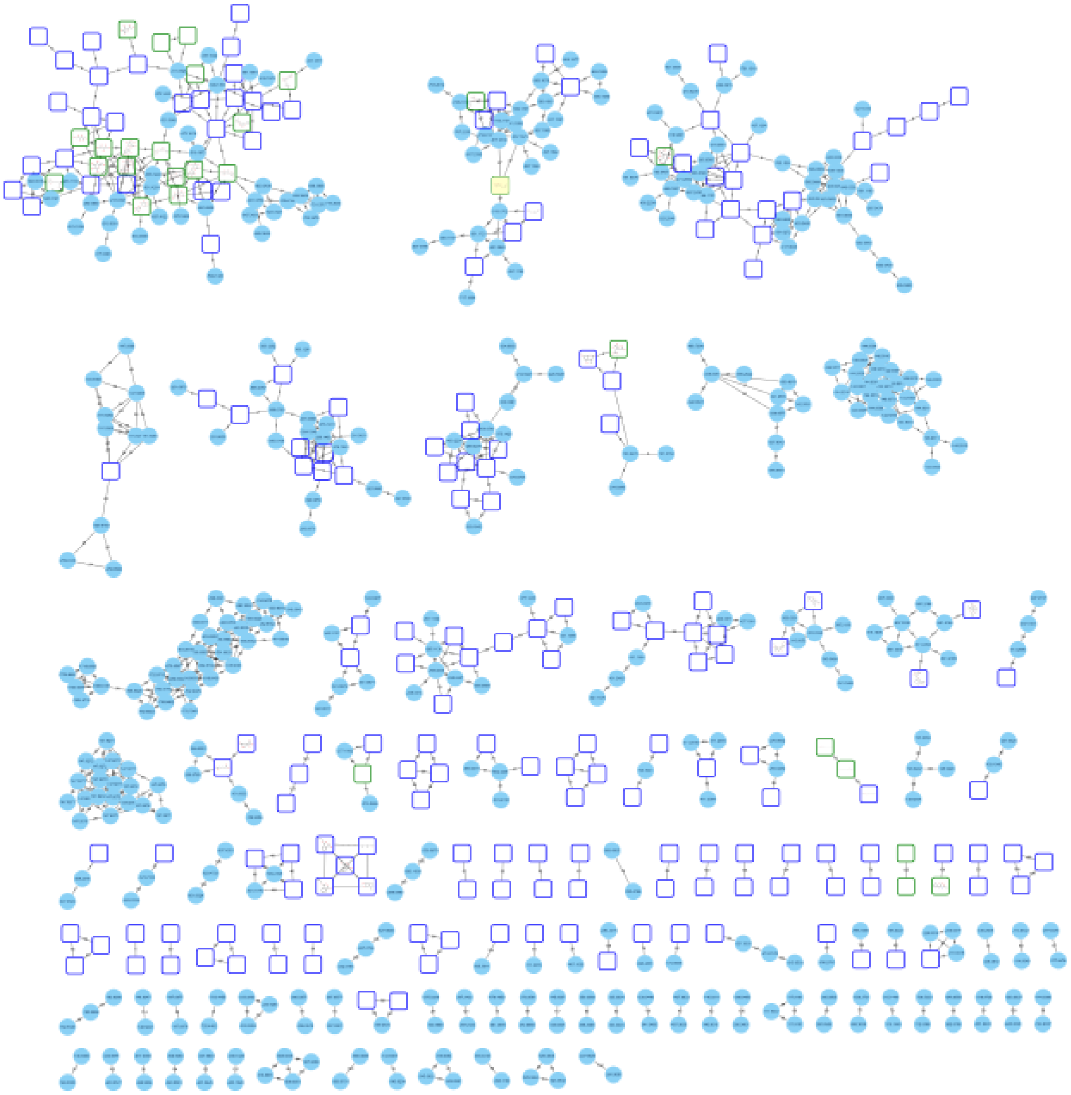

Figure S2. The entire NAP result of various *P. mume* extracts. Node borders in NAP are either green (spectral library match) or blue (*in silico* prediction).

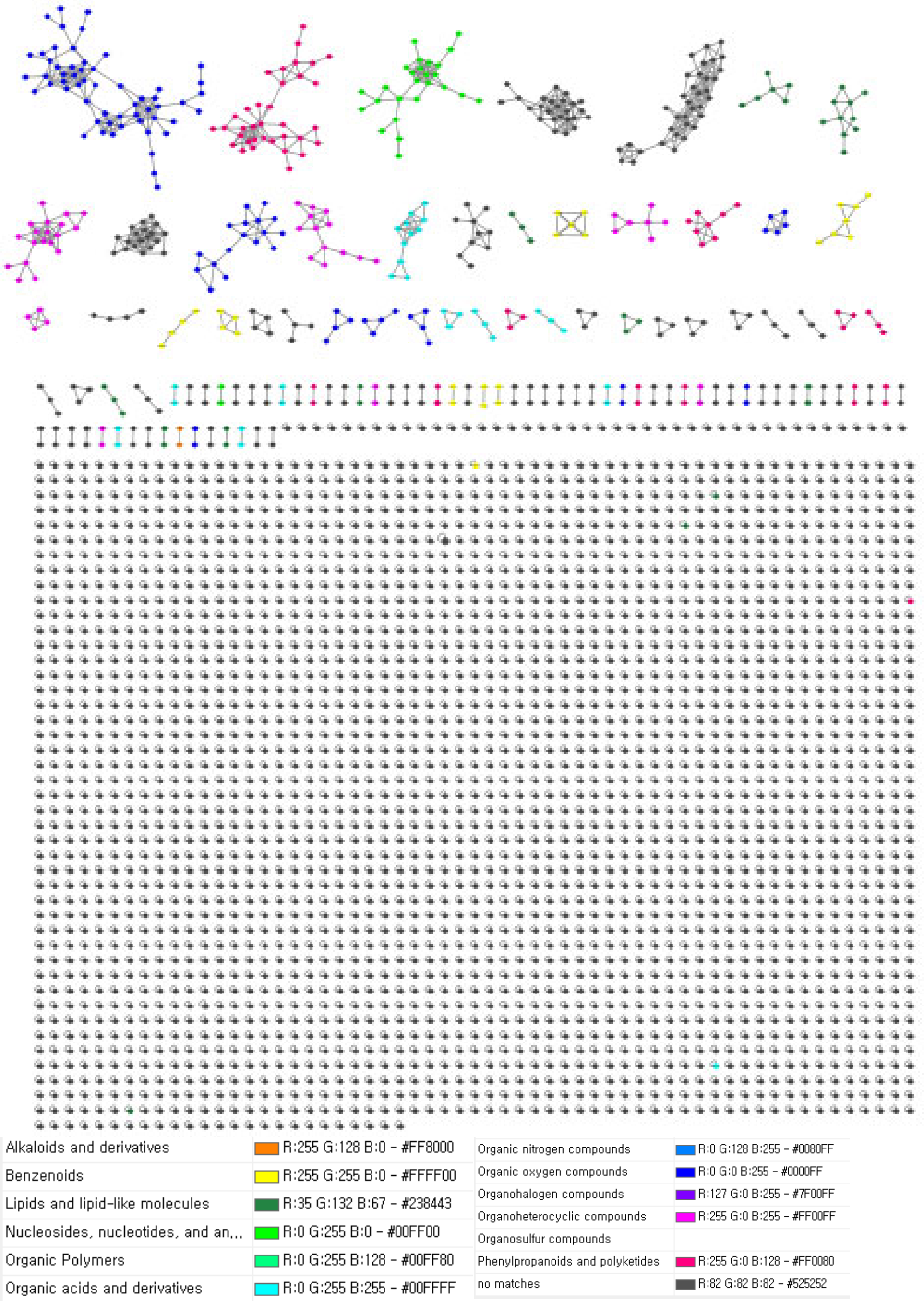

Figure S3. The entire MolNetEnhancer result of various *P.mume* extracts.

UPLC-UV chromatograms of MSE, FMSE, MSS, FMSS extracts (negative, 210 nm)

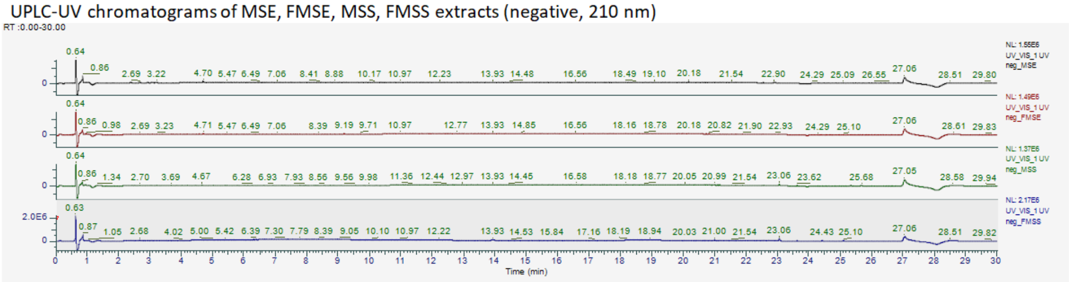

UPLC-UV chromatograms of MSE, FMSE, MSS, FMSS extracts (negative, 254 nm)

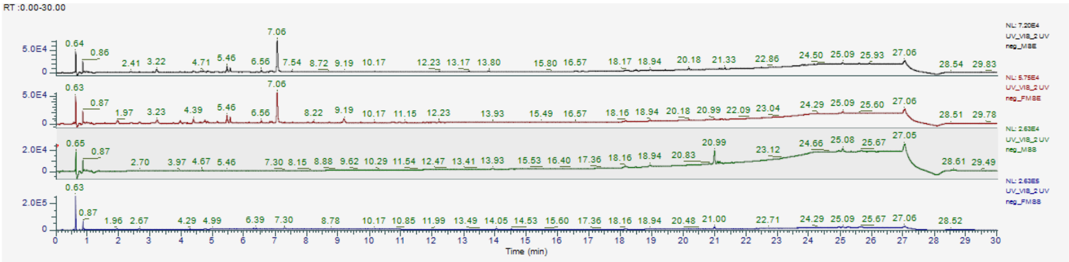

UPLC-UV chromatograms of MSE, FMSE, MSS, FMSS extracts (negative, 280 nm)

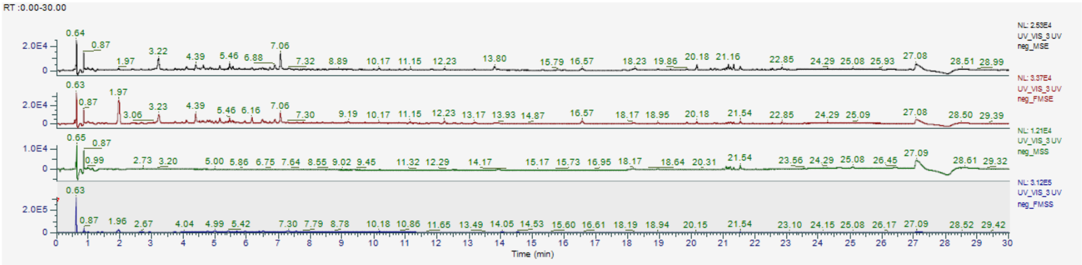

UPLC-UV chromatograms of MSE, FMSE, MSS, FMSS extracts (negative, 365 nm)

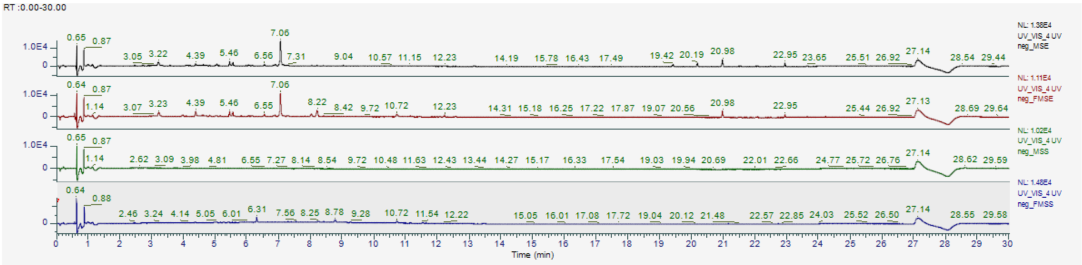

UPLC-MS base peak ion (BPI) chromatograms of MSE, FMSE, MSS, FMSS extracts (negative)

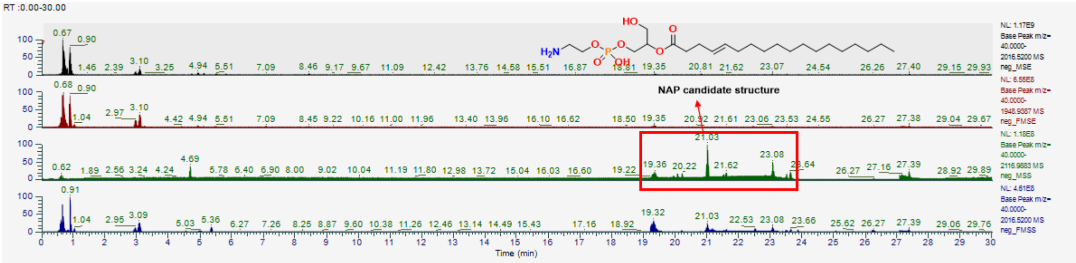

Figure S4. UPLC-UV-MS chromatograms of various *P. mume* extracts.

Table S1. Compounds list of candidates with MQ score > 0.7 obtained from. the FBMN networking analysis at GNPS.

retention time	m/z	compound name	cluster index (GNPS)
0.6001	225.0616	α-D-glucopyranose	142
0.6048	181.0718	galactitol	66
0.6048	209.0305	mucic acid	11985
0.6245	387.1138	α,α-trehalose	12
0.642	177.0407	gluclonic acid gamma-lactone	810
0.7229	387.1138	melibiose	53585
0.7681	341.1083	trehalose	167799
0.9402	188.0563	N-acetylglutamate	28027
1.3281	169.0142	gallic acid	167430
4.135	337.0934	(1R,3R,4S,5R)-1,3,4-trihydroxy-5-[(E)-3-(4-hydroxyphenyl) prop-2-enoyl]oxycyclohexane-1-carboxylic acid	167420
4.6067	517.1546	(E)-3-(4-hydroxy-3-methoxyphenyl)prop-2-enoate	167584
4.6636	367.1034	(1R,3R,4S,5R)-1,3,4-trihydroxy-5-[(E)-3-(4-hydroxy-3-methoxyphenyl) prop-2-enoyl]oxycyclohexane-1-carboxylic acid	6871
4.6928	456.151	amygdalin	22
4.7314	269.103	3,14-dioxo-8,19-bis({[(2S,3R,4S,5S,6R)-3,4,5-trihydroxy-6-(hydroxymethyl)oxan-2-yl]oxy}) -4,9,15,20-tetraoxatricyclodocosa-6,10,17,21-tetraene-11,22-dicarboxylate	168079
5.1934	191.0562	2-hydroxy-1,2,3-propanetricarboxylic acid	168544
5.2616	387.1664	fatty acyl hexoside	167625
5.3268	461.1658	benzyl 6-O-(6-deoxy-alpha-L-mannopyranosyl)-beta-D-glucopyranoside	100992
5.514	294.0983	prulaurasin	2674
6.6167	206.0824	N-acetylphenylalanine	3618
6.7381	521.2021	2-[[7-hydroxy-1-(4-hydroxy-3-methoxyphenyl)-3-(hydroxymethyl)-6-methoxy -1,2,3,4-tetrahydronaphthalen-2-yl]methoxy]-6-(hydroxymethyl)oxane-3,4,5-triol	172355
6.7589	787.0984	1,2,3,6-tetragalloylglucose	167824
7.255	303.0513	(2R,3R)-2-(2,6-dihydroxyphenyl)-3,5,7-trihydroxy-2,3-dihydrochromen-4-one	167992
8.4545	187.0978	azelaic acid	54
8.6993	287.0565	(2S,3S)-3,5,7-trihydroxy-2-(4-hydroxyphenyl)-2,3-dihydrochromen-4-one	167585
9.7401	693.2044	α-D-glucopyranoside	167532
9.8395	493.2283	6-O-[(2R,3R,4R)-3,4-dihydroxy-4-(hydroxymethyl)tetrahydro-2-furanyl]-beta-D-glucopyra-noside	194419
10.202	263.1291	abscisic acid	167506
20.7454	452.278	1-palmitoyl-2-hydroxy-sn-glycero-3-phosphoethanolamine	844
21.057	478.2938	PE(18:1/0:0)	7043
21.0594	571.2869	1-hexadecanoyl-sn-glycero-3-phospho-(1'-myo-inositol)	780

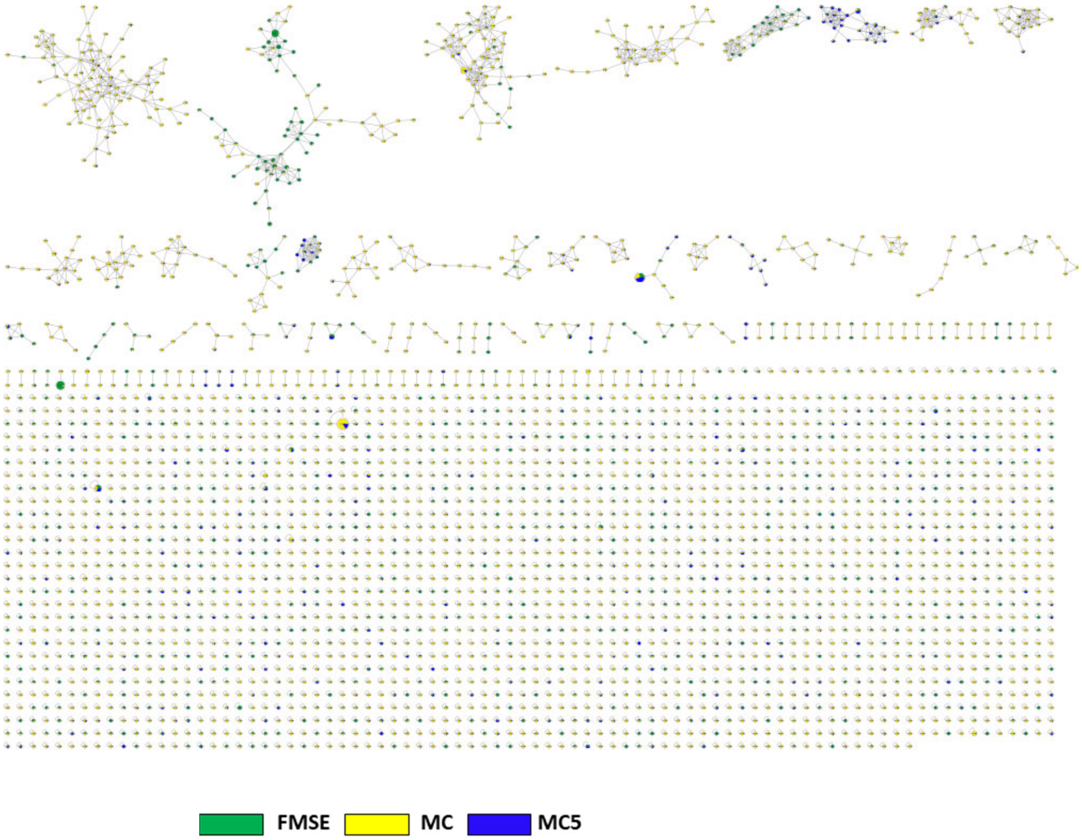

Figure S5. The entire feature-based molecular networking analysis of FMSE extract, MC fraction, and MC5 subfraction.

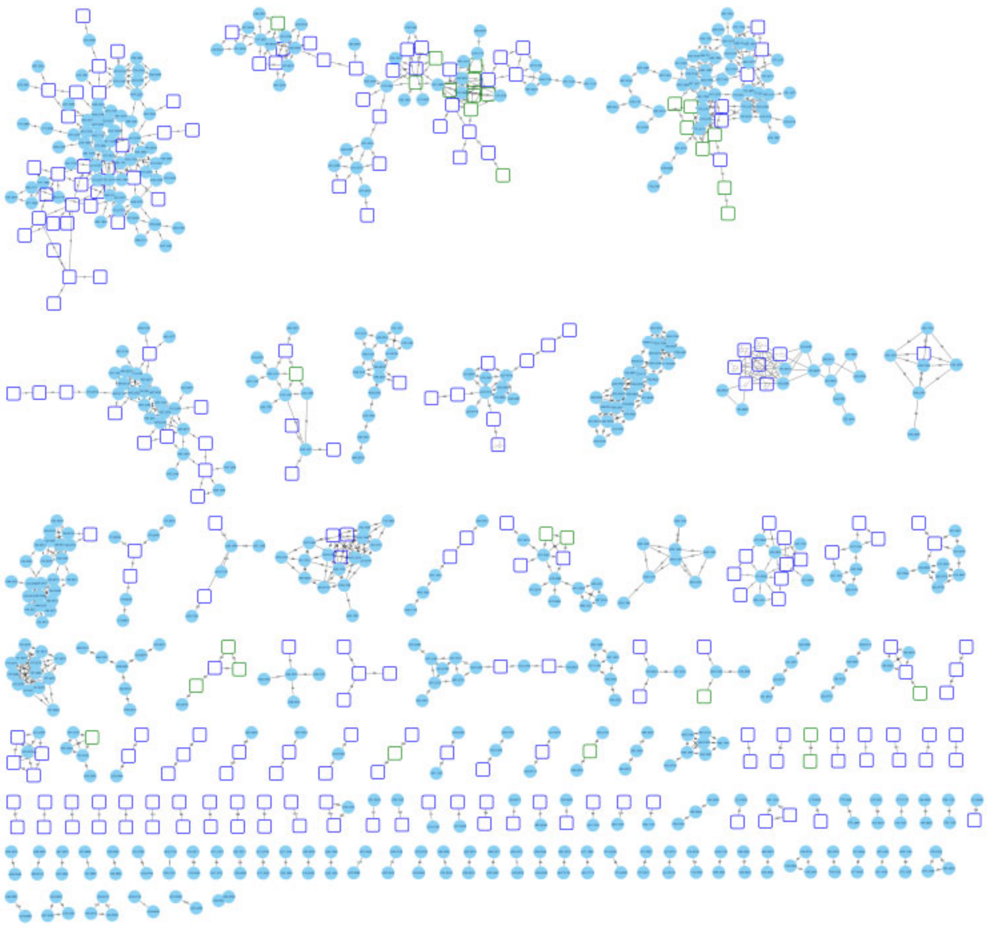

Figure S6. The entire NAP result of FMSE extract, MC fraction, and MC5 subfraction. Node borders in NAP are either green (spectral library match) or blue (*in silico* prediction).

Cluster Index	Parent Mass	RT Mean	LibraryID	FusionID	ConsensusID	MetFragID	CompleteRes
35	193.0506	9.5043	N/A		G S F	G S F	Link
592	193.0506	8.7412	N/A		G S F	G S F	Link
663	193.0507	11.2106	N/A		G S F	G S F	Link
77030	193.0507	6.9115	N/A		G S F	G S F	Link
165217	-193.0507	0.6093	gluconicacidCollisionEnergy:102040		G S F	G S F	Link
167108	193.0507	6.1998	N/A		G S F	G S F	Link

Showing 1 to 6 of 6 entries (filtered from 774 total entries) Previous 1 Next

Show 10 entries Search:

Identifier	MonoisotopicMass	Superclass	Class	NoExplPeaks	MCSS	Score	Fusion	Consensus
CCMSLIB00000221735	194.0579			1	G1	1.0000000		0.997
CCMSLIB00000426745	194.0579	Phenylpropanoids and polyketides	Cinnamic acids and derivatives	1	G1	1.0000000		1
CCMSLIB00000841621	194.0579			1	G2	1.0000000		0.936
CCMSLIB00000079831	194.0579			1	G3	0.8064641		0.943
CCMSLIB00004716419	194.0579	Organoheterocyclic compounds	Isocoumarans	1	G3	0.8064641		0.939
CCMSLIB00000569248	194.0579			0	G2	0.0000000		0.846

Showing 1 to 6 of 6 entries Previous 1 Next

Node 77030 MetFrag candidates

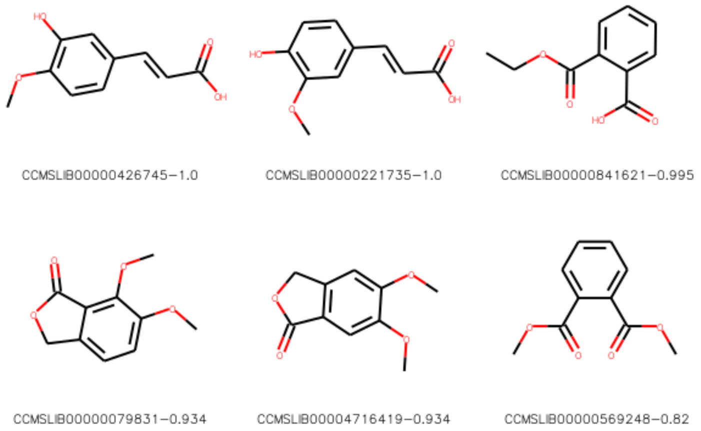

Figure S7. NAP viewer result for node 77030.

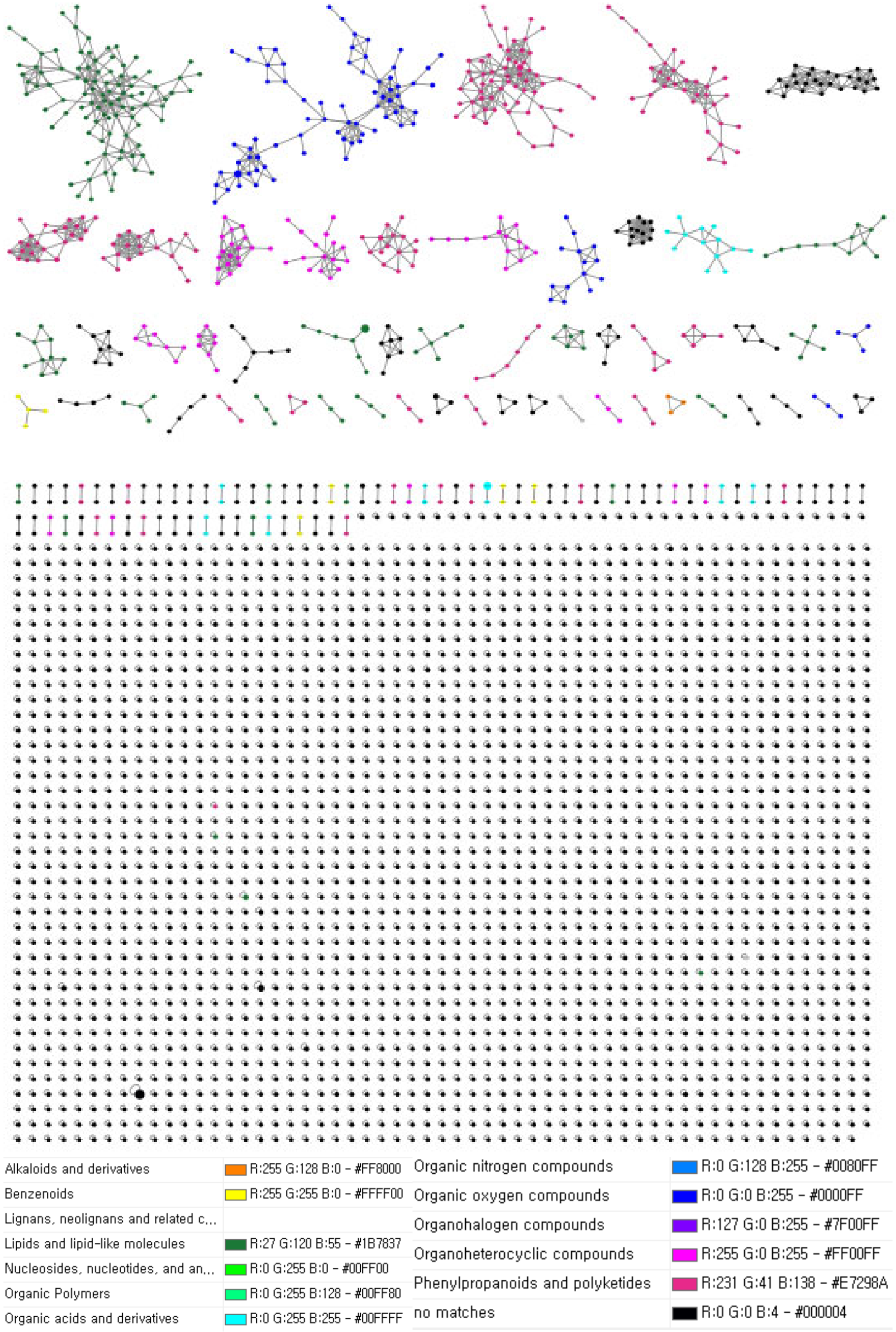

Figure S8. The entire MolNetEnhancer result of FMSE extract, MC fraction, and MC5 subfraction.

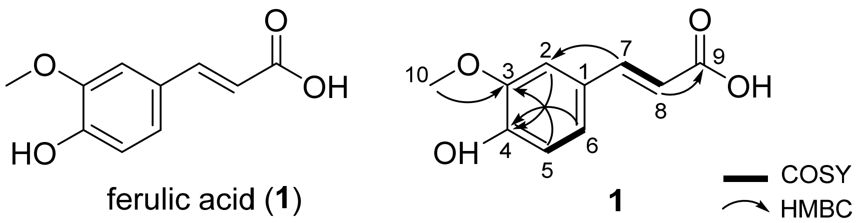

Figure S9. The planar structure and 2D NMR correlations of compound **1**.

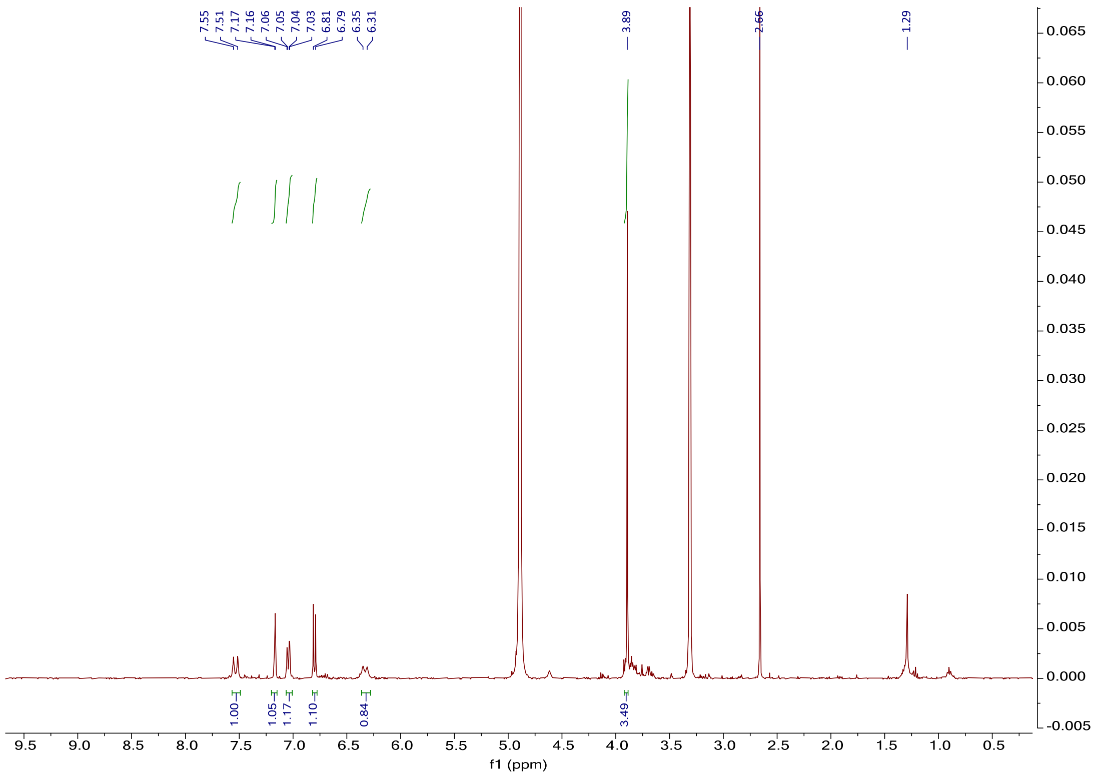

Figure S10. ^{1}H NMR (400 MHz, MeOH-d_4) spectrum of **1.**

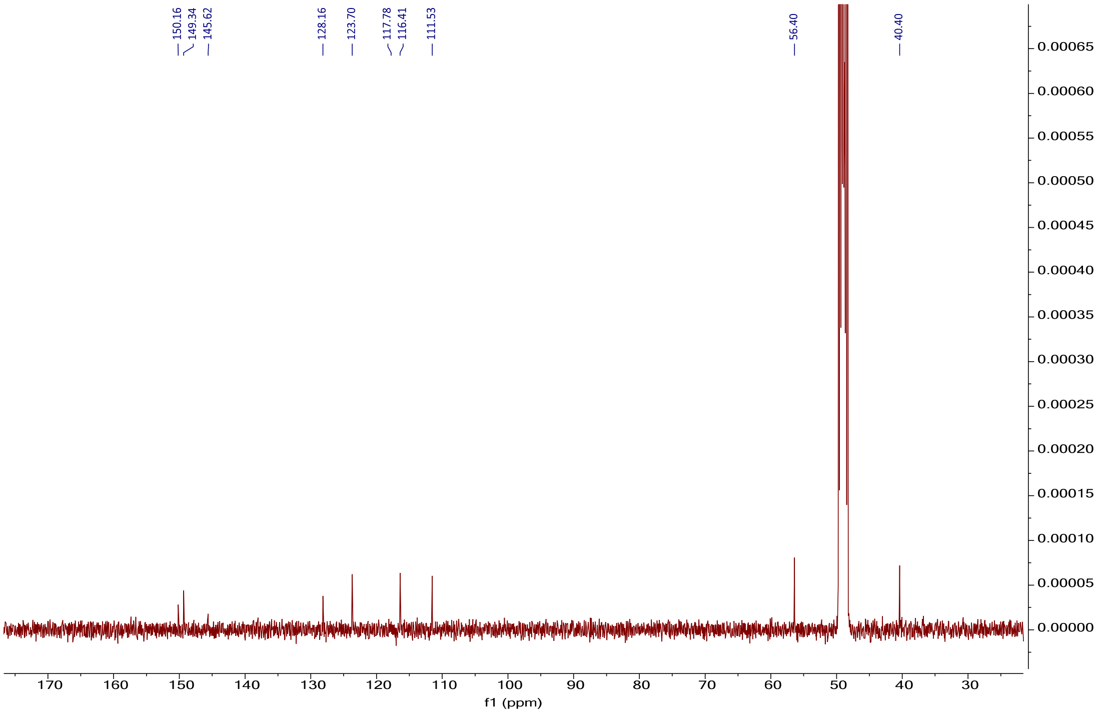

Figure S11. ^{13}C NMR (100 MHz, MeOH-d_4) spectrum of **1.**

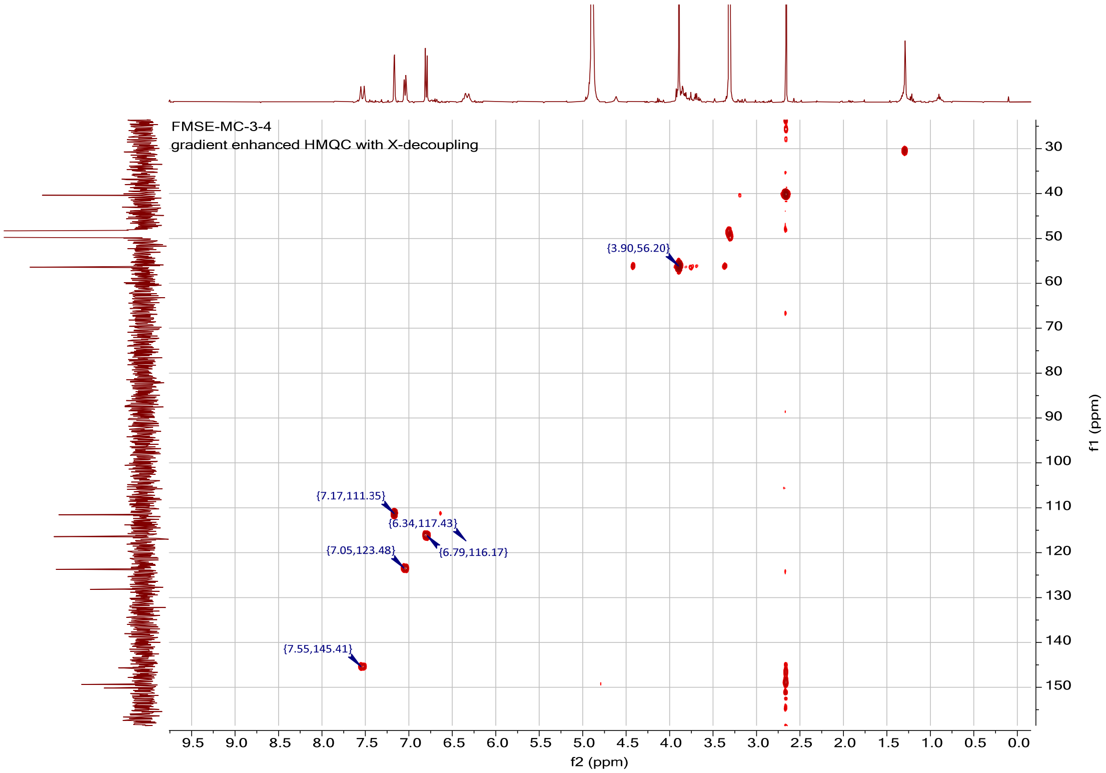

Figure S12. HSQC NMR (400 MHz, MeOH-d_4) spectrum of **1.**

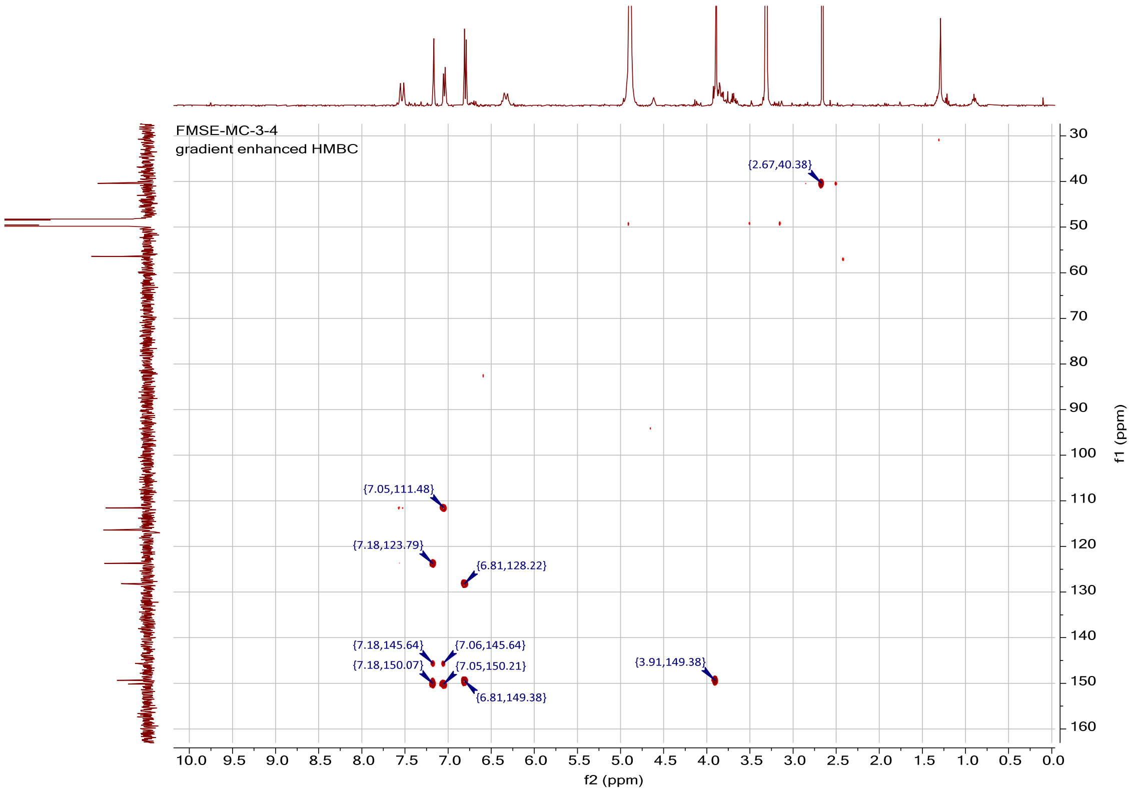

Figure S13. HMBC NMR (400 MHz, MeOH-d_4) spectrum of **1.**
